# Study on the central neural pathway and the relationship between the heart and small intestine via a dual neural tracer

**DOI:** 10.1371/journal.pone.0277644

**Published:** 2022-11-22

**Authors:** Fan ZHANG, Li-bin WU, Ling HU, Zi-jian WU, Shuai CUI, Qing YU, Rong-lin CAI

**Affiliations:** 1 Anhui University of Chinese Medicine, Hefei, Anhui Province, China; 2 Acupuncture and Meridian Research Institute, Anhui Academy of Chinese Medicine, Hefei, Anhui Province, China; 3 Key Laboratory of Xin’an Medicine, Ministry of Education, Anhui University of Chinese Medicine, Hefei, China; Georgia State University, UNITED STATES

## Abstract

Despite very different functions, studies increasingly report that there may be a potential central nervous anatomical connection between the heart and the small intestine. In this study, the central nervous anatomical relationship between the heart and small intestine was studied via a viral tracer. Pseudorabies virus (PRV) syngeneic strains with different fluorescent reporter genes (eGFP or mRFP) were microinjected into the heart walls and small intestinal walls of male C57BL/6J using glass microelectrode. The results showed that the co-labeled nuclei in the brain were lateral periaqueductal gray (LPAG) and ventrolateral periaqueductal gray (VLPAG) in the midbrain, mesencephalic trigeminal nucleus (Me5), and motor trigeminal nucleus anterior digastric Part (5Adi) in the pons. The co-labeled sites in the spinal cord were intermediolateral column (IML) in the second thoracic vertebra, IML and lamina 7 of the spinal gray (7SP) in the third thoracic vertebra, and IML in the fourth thoracic vertebra. Our data show that there is a neuroanatomical connection between the small intestine and the heart in the central nervous system (CNS). Neuroanatomical integration of the heart and small intestine may provide a basis for revealing the physiological and pathological interactions between the circulatory and digestive systems. The interactions may be mediated more effectively through sympathetic nerves.

## Introduction

The acquisition of multicellular bodies leads to a higher level of order in the evolution of cells, tissues and organs [1, 2], which necessitates a parallel development communication systems between these organs-organs (anatomical entities) [3]. These communication systems include the neurons of the nervous system and numerous soluble mediums [4]. Thus, communications between organs are involved in the majority physiological and pathological events [5], and many diseases originate from impaired communication between organs. In addition, damage to a single organ can also lead to pathological phenotype in distant organs [6].

It has been known since ancient times how organs communicate with one another. Galen (129–217), the father of experimental physiology, had originally hypothesized and experimentally proved the physiological role of inter-organ communication [7, 8]. Investigating inter-organ communication has garnered more attention in recent years. The mechanisms to control the body’s metabolic balance have been emphasized in a number of groundbreaking research, including the liver-intestine connection [9], kidney-lung connection [10] and heart-lung connection [11]. With the evolution of multicellular organisms, the interactions between peripheral organs and the central nervous system have diversified. The physiology of the whole organism has undergone a revolution as a result of significant new discoveries like the intestine-brain axis [12], the heart-brain axis [13], the intestine-lung axis [14], and the heart-intestine axis [15]. At the same time, our study found that small intestine ligation could result in cardiac enlargement, myocardial degeneration, and other pathological changes [16]. Neuronal connections are the key messengers for inter-organ communication and the material basis of organ-brain communication [17], As an illustration, the intestine nerve and the vagus nerve form a special physical connection in the brainstem [18].

**Fig 1:**
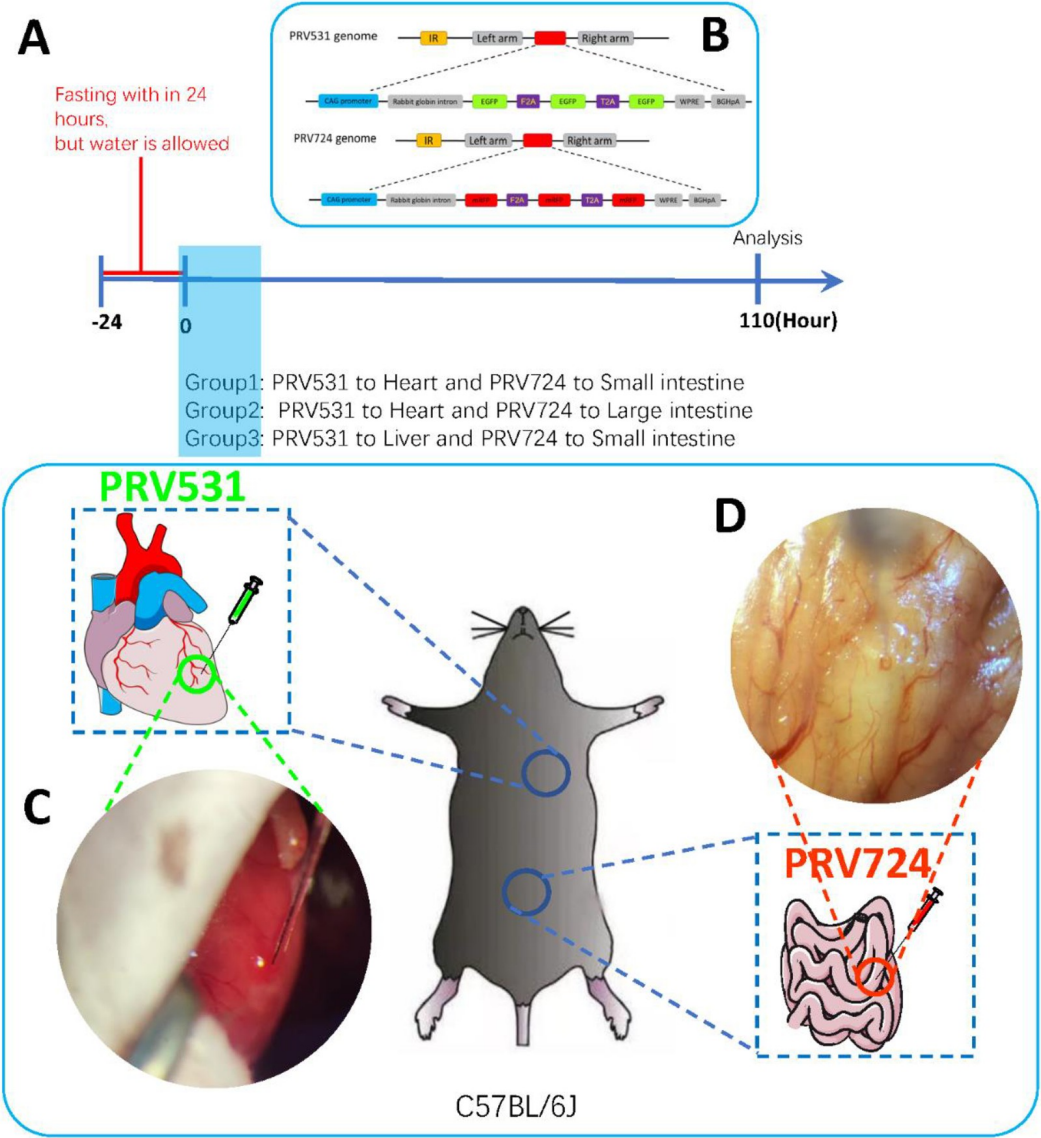
Experimental setup and design. **A)** Protocol. **B)** Genomes of PRV531 and PRV724. **C)** PRV531 was injected into the left ventricular wall of the heart (2 ul 0.8 ul/min). **D)** PRV724 was injected into the wall of small intestinal (2 ul 0.2 ul/min).

In reality, many neural pathways of organ-to-organ connections have not yet been discovered, and the dual-virus tracing method will broaden our understanding of the organ-organ communication neural circuit. In the organ-brain communication pathway, we try to find the unique physical connection between the heart and small intestine in the brain, and provide some potentially innovative targets. Specifically, that neurons in the brain regions associated with cardiovascular and digestive function have neuroanatomical connections to permit coordination of multiple and complementary responses in these downstream organs. In order to carry out this research, we use the retrograde transneuronal viral tracing technical. Pseudorabies virus (PRV) syngeneic strains with different fluorescent reporter genes (eGFP or mRFP) were injected into the heart walls and small intestinal walls of male C57BL/6J mice (Fig 1). The PRV is transported retrograde across neurons in a time-dependent manner; it infected neurons and expressed red or green fluorescent proteins. We expect to observe the possible neuroanatomical co-localization of the heart and small intestine in the center via a dual neural tracer.

## Materials and methods

### Animals

C57BL/6J mice (20–25 g) were purchased from the Chinese Academy of Sciences Wuhan Institute for Experimental Animals. Twelve male mice were randomly divided into three groups: heart-small intestine group (heart and small intestine were injected with PRV531 and PRV724 respectively), heart-large intestine group (heart and large intestine were simultaneously injected with PRV531 and PRV724, respectively), and liver-small intestine group (liver and small intestine were injected with PRV531 and PRV724 respectively). There were 4 mice in each group. The mice were housed under 12-hour light/dark cycle (up to 5 per cage), and water and food were given free. All experimental procedures have been approved by the Animal Nursing and Utilization Committee of the Anhui University of Chinese Medicine and were carried out in accordance with the Agency Animal Welfare Guide.

### Virus

PRV531 (PRV-CAG-EGFP) and PRV724 (PRV-CAG-mRFP) were provided by F. Xu (Wuhan, China); titers were estimated at 7*10^9^ genomic copies/mL.

Preparation of Recombinant Virus PRV531 and PRV724 [19]. First, Preparation of plasmid PS506[pcDNA3.1(+)-left arm-Ubc-3×EGFP-WPRE-bGHpA-right arm]. Second, rabbit β-globin intron was inserted into the plasmid PS506 treated with AsiSI to prepare the plasmid PS529. Third, The CAG was inserted into the plasmid PS529 treated with ClaI and AsiSI to prepare the plasmid PS531. Fourth, to construct the plasmid PS724, the fragment mRu-by3-F2A-mRuby3-T2A-mRuby3 was synthesized, which replaced the EGFP-F2A-EGFP-T2A-EGFP using the AsiSI and AgeI-treated plasmid PS531. And finally, PS531 expression cassette and PS724 expression cassette were inserted into the middle of the gG gene of the PRV Bartha genome respectively to obtain PRV531 and PRV724.

### Surgery and viral injections

Mice were fasted for 24 h, but water was allowed. Then mice were anesthetized by intraperitoneal injection of 1% pentobarbital sodium (10 mL/kg). After fixing the upper teeth and limbs, mice were placed on a heating pad to maintain the body temperature at 36°. The depilation agent was used to remove the hair on the neck of mice, which was subsequently irradiated using a cold light source. A tweezer was put into the mouth of mice and stretched out. After inserting the indwelling needle (18G 1.3*30 mm 70 ml/min) into the trachea through the vocal cord, the needle core was pulled out and connected to a ventilator (respiratory ratio 1:1, tidal volume 1–1.5ml, respiratory frequency 120-130/min).

Heart injection of PRV531: After routine surgical disinfection, a 4-6mm vertical incision was performed on the left chest. The sternum was exposed after the superficial pectoralis muscle and the anterior serratus muscle were separated by microscopic tweezers. A mouse chest dilator opened the fourth and fifth intercostal spaces, and the heart was exposed through the opening and protruded outward. Heart stereotaxic injection was performed on the stereotactic frame (stoelting Stereotaxic Instrument, America). PRV531 was injected into the left ventricular wall of the heart (800 nl/min, 2 ul) using a Hamilton micro-syringe (the tip of the syringe is a glass microneedle). After injection, the injection needle was fixed in place for 5 min. Immediately after the needle was removed, a cotton cloth soaked with disinfectant was used to absorb any outflow from the injection site to the surface of the heart. After operation, the skin layers and muscle were sutured, and iodine was used to disinfect the operation area.

Small intestine injection of PRV724: Depilation cream was used to remove body hair from the abdomen of the mice. The small intestine was then exposed through the incision in the middle of the abdomen and protruded outward. A small intestine stereotaxic injection was administered on the stereotactic frame (stoelting Stereotaxic Instrument, America); PRV724 was injected into the lateral part of the wall of the small intestine (200 nl/min, 2 ul) using a Hamilton micro-syringe.

### Perfusion, section, and imaging examination

Four mice were sacrificed 110 h later to retrieve the spinal cord and brain. Each animal was deeply anesthetized, and 0.9% cold saline and 4% paraformaldehyde (PFA) were perfused through the heart. The brains and spinal cords were collected from each animal and then placed in 4% PFA until sectioning. We sectioned brains at 50 μm and stained with DAPI for 10 minutes at room temperature.

Images were acquired using an Olympus VS120 microscope(Japan) and an Olympus FV1000 confocal microscope(Japan). Images were analyzed using Image-Pro Plus 6.0 and Image J software (version Fiji). Colocalization analysis of the neurons used fluorescence intensity. 3D neuron reconstruction used Imaris Software. Different brain regions were identified using the Allen Mouse Brain Atlas (https://mouse.brain-map.org/) and the Mouse Brain in Stereotaxic Coordinates (the Mouse Brain in Stereotaxic Coordinates 4th Ed., Academic, 2012).

### Statistical analysis

Statistical analysis was performed using GraphPad Prism v7.0 (GraphPad Software) and IBM SPSS Statistics 23.0. The 30 mm thick brain and spinal cord sections were collected from 12 samples. ImageJ software was used for the quantification of fluorescence intensity and amount of neurons. Two samples were taken from each nuclear of each mouse for statistical analysis (one-way ANOVA). The data were expressed by mean SEM, *P* < 0.05, indicating a significant difference.

## Result

PRV would be ingested, replicated, and transmitted across synapses by specific neuronal populations 110 h after inoculation with PRV on the heart and intestinal walls of normal mice. Studies proved that intravenous PRV did not cause neuronal infection [20]. Therefore, we found that all viruses in the brain and spinal cord were specifically infected by the heart and small intestine. Our results revealed neuroanatomical integration of the small intestine and heart in the spinal cord and brain. To put it simply, a close neuroanatomical relationship was found between the gastrointestinal system and the cardiovascular system, which seemed to be closely related to the sympathetic nerve. The output of the intestinal sympathetic nerve was integrated into the segments of the T2-T4 spinal cord related to the heart. Interestingly, the integration of neurons was also found in the nuclei related to the splanchnic autonomic nervous system in the brain, suggesting a close relationship between the two systems at the neuroanatomical level and the leading role of the regulation of sympathetic nerves in this connection.

### Common labeling in the brain

**Fig 2:**
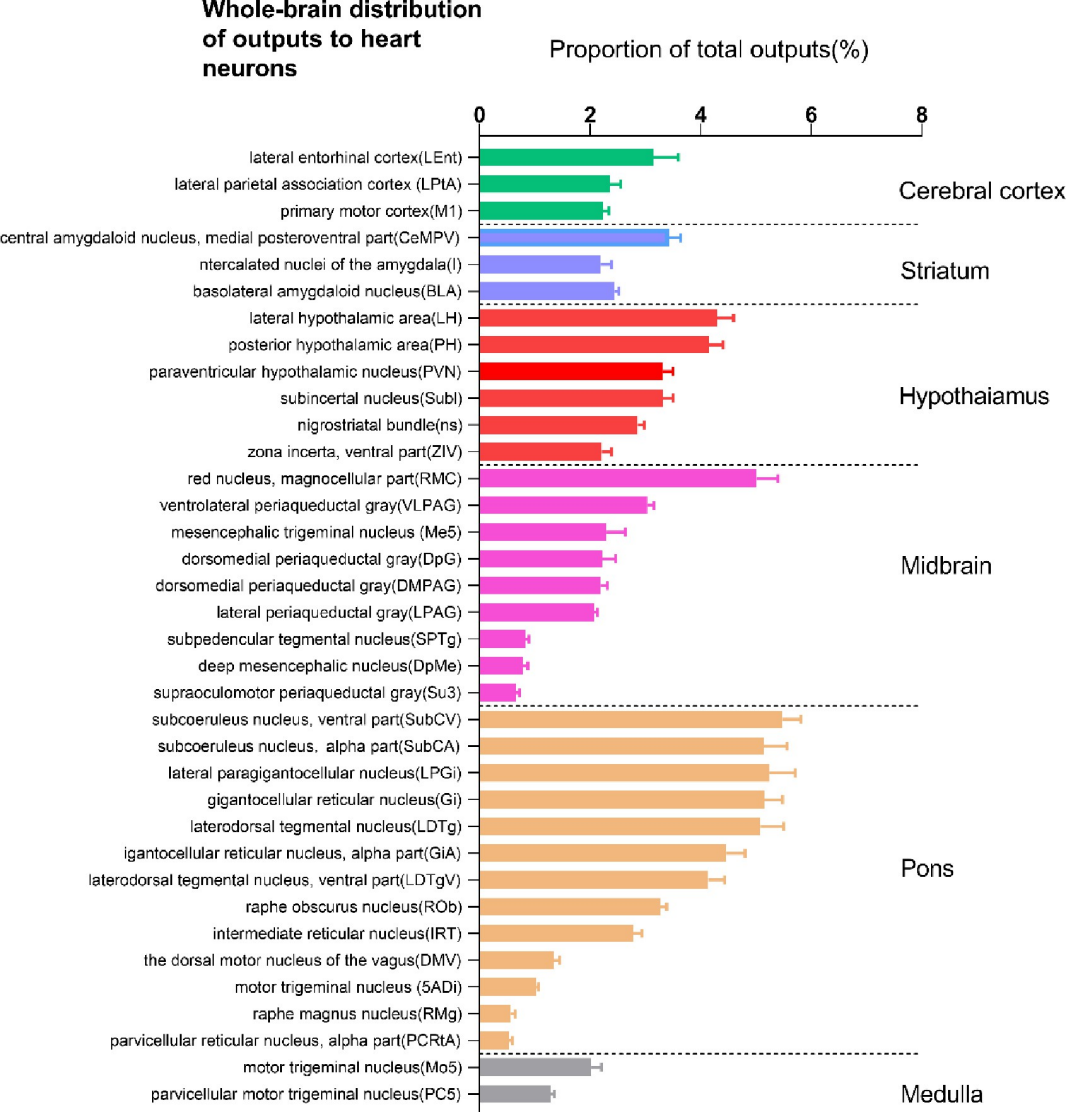
Statistical analysis of the whole-brain distribution of outputs to heart. This data is based on the ratio of the number of EGFP-expressing neurons in each nucleus to the total number of labeled neurons in each brain (n = 4 mice). The percentages reflect the retro-labeled output neurons from 36 regions in C57/BL mice. Brain areas are grouped into six general structures: cerebral cortex, hypothalamus, midbrain, pons, and medulla.

**Fig 3:**
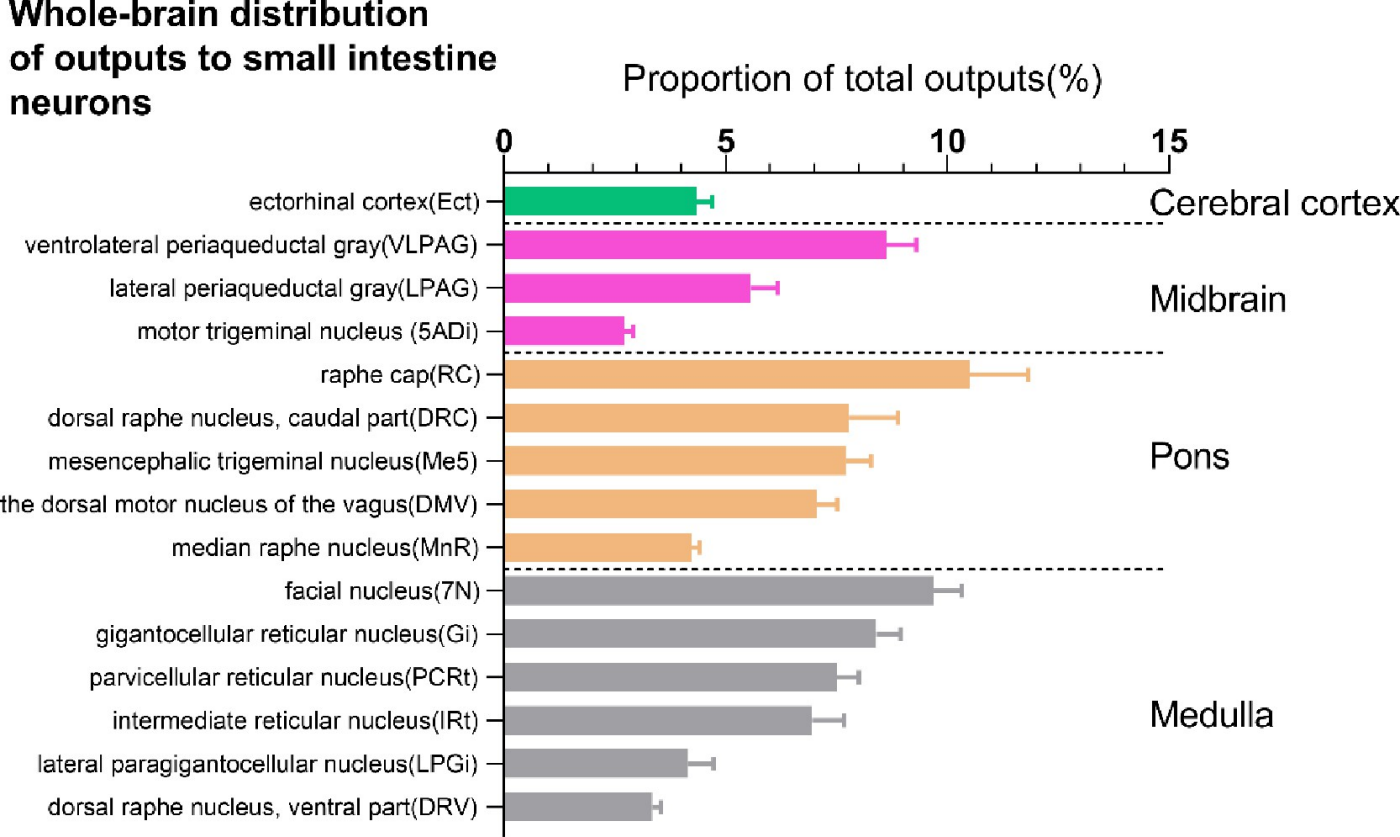
Statistical analysis of the whole-brain distribution of outputs to small intestine. These data are based on the ratio of the number of mRFP-expressing neurons in each nucleus to the total number of labeled neurons in each brain (n = 4 mice). Percentages report retro-labeled output neurons from 15 regions in C57/BL mice. Brain areas are grouped into four general structures: cerebral cortex, midbrain, pons, and medulla.

We constructed a list of the distribution of output nuclei from the whole brain to the heart (based on the ratio of the number of EGFP-expressing neurons in each nucleus to the total number of labeled neurons in each brain, Fig 2, n = 4). Our study shows that there are neuroanatomical connections between 36 nuclei and the heart. And we constructed a list of the whole brain output of the small intestine (based on the ratio of the number of mRFP-expressing neurons in each nucleus to the total number of labeled neurons in each brain (Fig 3, n = 4). Our study shows that there are neuroanatomical connections between 15 nuclei and the small intestine.

**Fig 4:**
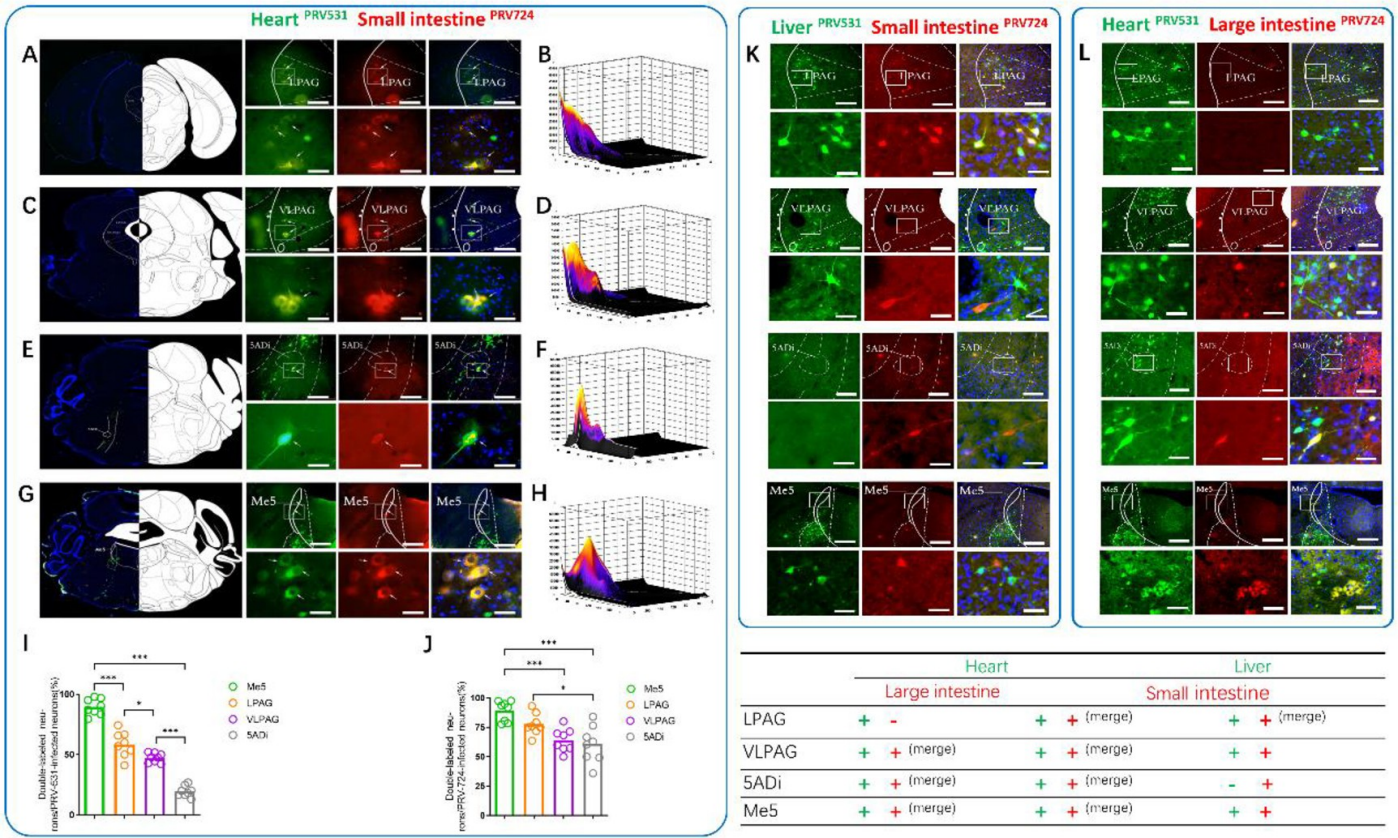
The common output of the whole-brain to small intestine and heart. **A/C/E/G)** Upper, Heart (green) neurons co-labeled with Small intestine (red) in the LPAG/VLPAG/5Adi/Me5 (scale bar, 100 um). Lower, magnification images of the area outlined by the white box (scale bar, 25 um). **B/D/F/H)** Colocalization analysis of neurons co-labeled in LPAG/VLPAG/5Adi/Me5. **I)** Analysis of co-localized neurons (double-labeled neurons/PRV-531 infected neurons). **J)** Analysis of co-localized neurons (double-labeled neurons/PRV-724 infected neuron). **K)** Upper, Liver (green) neurons co-labeled with Small intestine (red) in the LPAG/VLPAG/5Adi/Me5 (scale bar, 100 um). Lower, magnification images of the area outlined by the white box (scale bar, 25 um). **L)** Upper, Heart (green) neurons co-labeled with Large intestine (red) in the LPAG/VLPAG/5Adi/Me5 (scale bar, 100 um). Lower, magnification images of the area outlined by the white box (scale bar, 25 um). The white arrows show the neural of colocalization fluorescence labeling; yellow fluorescence shows colocalization of green and red fluorescence; LPAG, lateral periaqueductal gray; VLPAG, ventrolateral periaqueductal gray; 5Adi, motor trigeminal nucleus anterior digastric Part; Me5, mesencephalic trigeminal nucleus. All data are presented as the mean ± SEM.***P<0.001, **P<0.01, *P<0.05.

We observed co-labeled neurons in VLPAG, LPAG, Me5, and 5ADi (Fig 4 and S1 Video https://figshare.com/s/df5171d4f2bd8ed97ddd DOI: 10.6084/m9.figshare.15098250). We also analyzed the co-labeled neurons (double-labeled neurons/PRV-531-infected neurons and double-labeled neurons/PRV-724-infected neurons). We found that 58.60%±3.71% of the PRV531-infected neurons in LPAG were infected by PRV724, 47.36%±1.37% of the PRV531-infected neurons in VLPAG were infected by PRV724, 89.54%±2.37% of the PRV531-infected neurons in Me5 were infected by PRV724, 19.58%±1.61% of the PRV531-infected neurons in 5ADi were infected by PRV724; Me5>LPAG (P<0.001), LPAG>VLPAG (P<0.05), VLPAG>5Adi (P<0.001) (Fig 4, n = 4). In addition, 77.66%±3.23% of the PRV724-infected neurons in LPAG were infected by PRV531, 64.24%±3.45% of the PRV724-infected neurons in VLPAG were infected by PRV531, 89.05%±3.05% of the PRV724-infected neurons in Me5 were infected by PRV531, 60.69%±5.49% of the PRV724-infected neurons in 5ADi were infected by PRV531; Me5>VLPAG (P<0.001), LPAG>5ADi (P<0.05) (Fig 4, n = 4). There is no common neuroanatomical connection between the liver and the small intestine in VLPAG, 5Adi, Me5. Heart and large intestine neuroanatomical link not found in LPAG.

### Common labeling in the spinal cord

**Fig 5:**
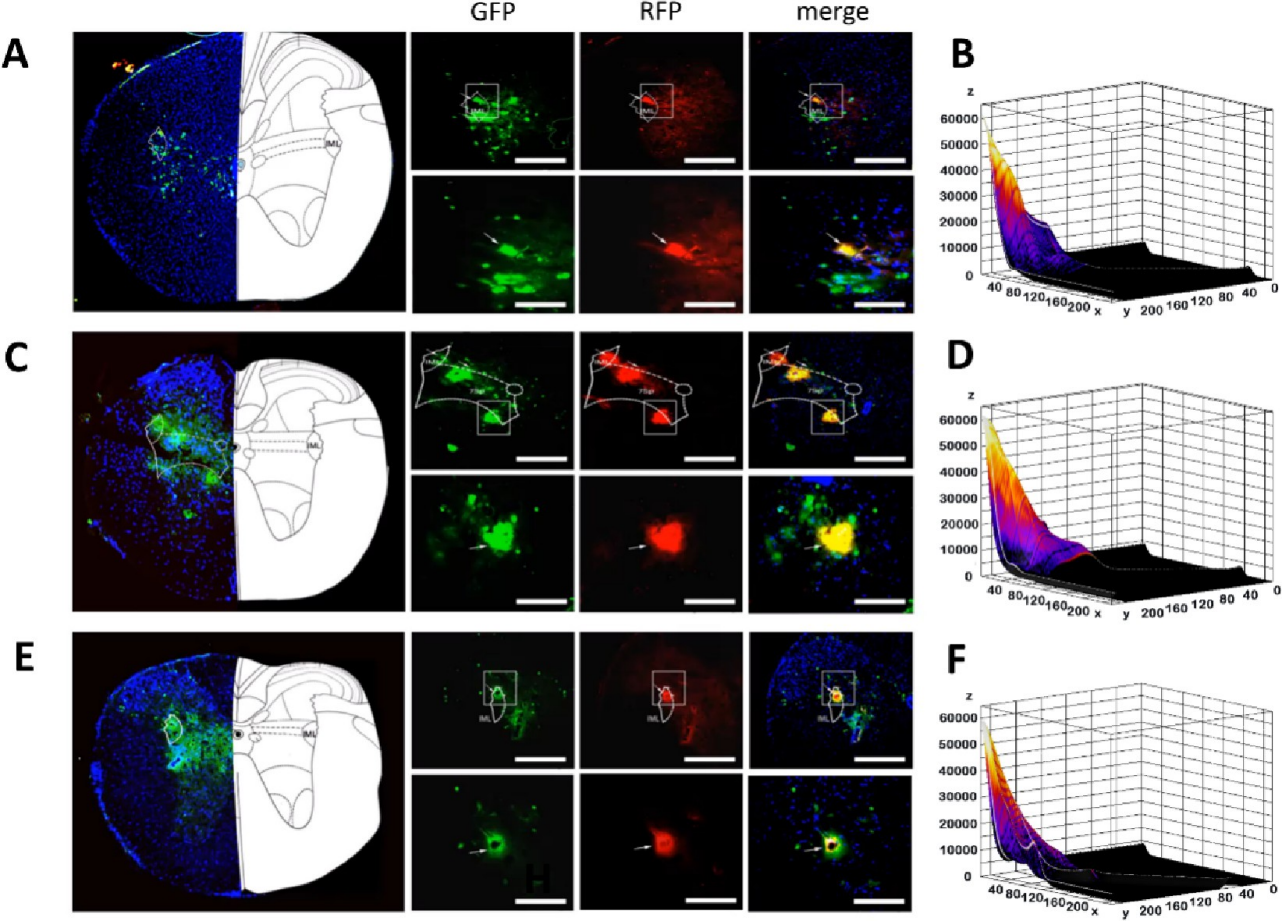
Common labeling in the spinal cord. **A)** Upper, PRV531 (green) neurons co-labeled with PRV724 (red) in the IML (T2) (scale bar, 100 um). Lower, magnification images of the area outlined by the white box (scale bar, 50 um). **B)** Colocalization analysis of neurons co-labeled in the IML (T2). **C)** Upper, PRV531 (green) neurons co-labeled with PRV724 (red) in the IML, 7Sp (T3) (scale bar, 100 um). Lower, magnification images of the area outlined by the white box (scale bar, 50 um). **D)** Colocalization analysis of neurons co-labeled in the IML, 7Sp (T2). **E)** Upper, PRV531 (green) neurons co-labeled with PRV724 (red) in the IML (T4) (scale bar, 100 um). Lower, magnification images of the area outlined by the white box (scale bar, 50 um). **F)** Colocalization analysis of neurons co-labeled in the IML (T4). The white arrows show the neural of colocalization fluorescence labeling; Yellow fluorescence shows colocalization of green and red fluorescence; IML, intermediolateral column; 7Sp, lamina 7 of the spinal gray; All data are presented as the mean ± SEM.***P<0.001, **P<0.01, *P<0.05.

PRV531- and PRV724-labeled neurons were found in the cervical, thoracic, and lumbar segments mainly in the IML of gray matter of the spinal cord and the gray matter next to the central canal of the spinal cord. Common labeled neurons were found in the IML of T2, the IML and 7Sp of T3, and the IML of T4. Some evidence exists that the sympathetic preganglionic fibers of the heart leave the spinal cord at the T1-T4 level, and then synapses are formed in the left and right stellate ganglion and T2-T4 thoracic ganglion (Fig 5). Finally, the postganglionic fibers originate from these ganglia and reach around the sinoatrial node and the coronary sinus [21]. Our observations are consistent with the research reports above. In addition, the co-labeled neurons are oval or pear shaped indicating that the heart and small intestine connect in the spinal cord through sympathetic nerves [22]. Many clinical reports have shown that the sympathetic nerve is involved in the process of the change of heart rate variability in patients with irritable bowel syndrome [23–25]. Therefore, our results provide possible neuroanatomical evidence for the pathogenesis of irritable bowel syndrome.

## Discussion

In the past 10 years, people have been very interested in how the intestine and the brain communicate with each other. The steady state of the body requires fine-tuning of the communication system between organs, including the communication between the liver and the intestinal tract, the communication between the lungs and the heart [26], etc. Intestinal autonomic nerve function diseases are often accompanied by changes in cardiac autonomic nerve function. The brain communicates with viscera through multiple parallel pathways, including two branches with autonomic nerves, sympatho-adrenalmedullary (SAM) system (responsible for regulating gut-associated lymphoid tissue), and hypothalamus-pituitary-adrenal axis (HPA) [27]. In addition, as the key structures of visceral regulation, the amygdala and hypothalamus are integrated into different regions of the PAG of the midbrain [28]. PAG is a comprehensive site responsible for regulating many physiological functions, including cardiovascular regulation driven by the sympathetic nervous system [29] and digestive system regulation (such as intestinal homeostasis and food intake). The rostral ventrolateral medulla (RVLM) and ventromedial medulla (VMM) of the medulla oblongata receive projections from PAG and eventually regulate visceral homeostasis through sympathetic nerves [30]. The sympathetic innervation of the gastrointestinal tract and its role in regulating gastrointestinal function have been widely recognized and expressed as inhibitory regulation through cholinergic transmission [31]. Our study found that integration of intestinal and cardiac autonomic nervous regulation seemed to exist in PAG, indicating a close relationship between the heart and the intestinal tract. Meanwhile, Heart and intestine are specific for neuroanatomical integration in the brain.

**Fig 6:**
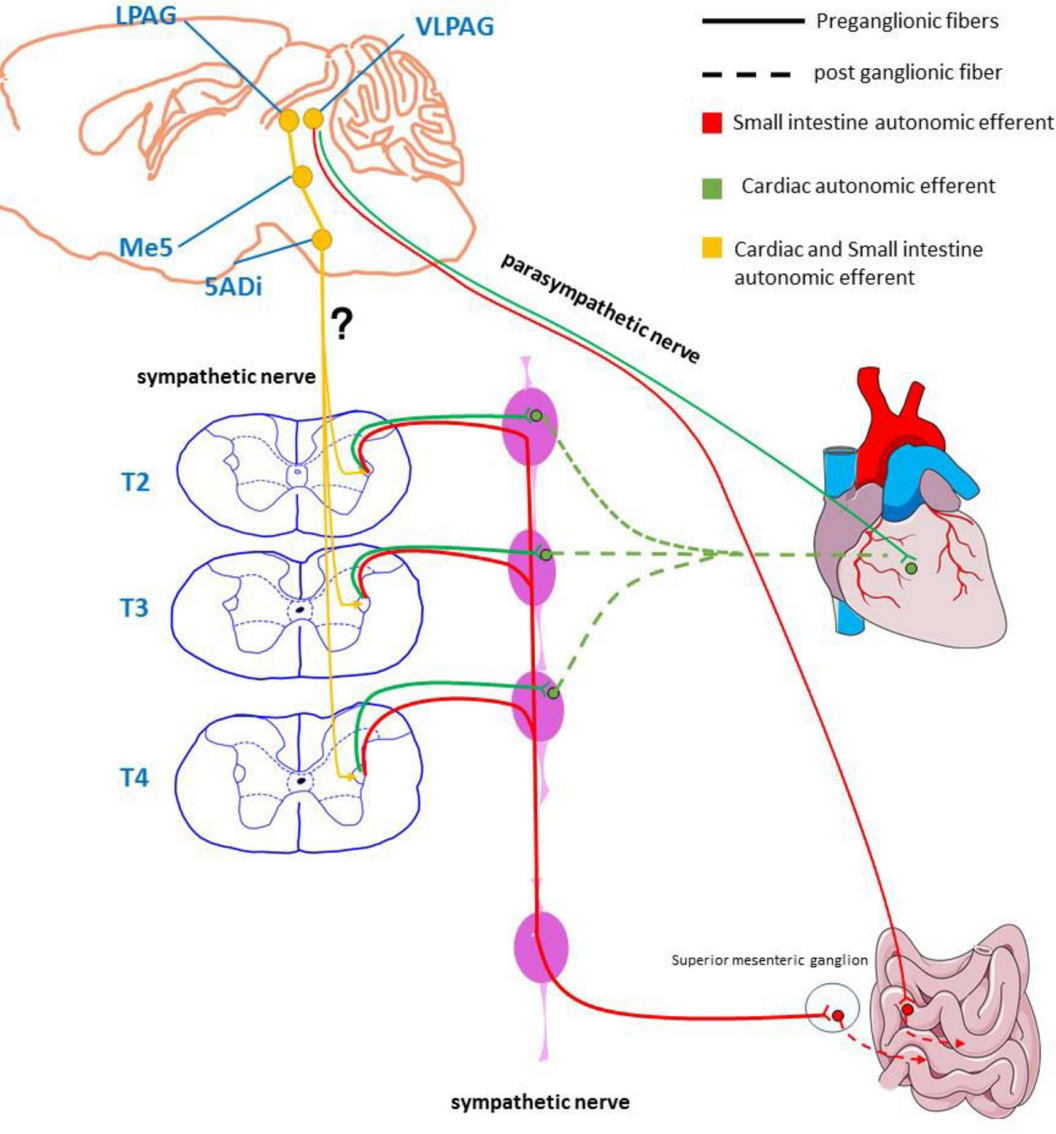
Hypothetical diagram of autonomic nerve connection between heart and small intestine. Schematic diagram of the autonomic nervous outflow from the central nervous system to heart and small intestine based on tracing studies using the pseudorabies virus (PRV).

In recent years, the importance of the brain-gut axis in gastrointestinal diseases has been increasingly recognized especially in irritable bowel syndrome, functional dyspepsia, etc. People have gradually realized that the neuroregulation of intestinal function not only depends on the inherent intestinal nervous system (ENS): The regulation of the CNS cannot be ignored. Studies have shown that patients with insulin-dependent diabetes may die of ventricular arrhythmias caused by overeating and insulin [32–35]. The mechanism is a disorder of autonomic nervous regulation in the small intestine (mainly characterized by increased vagal tone) [36]. It can reduce the elevation of the ST segment and inhibit spontaneous ventricular fibrillation by stimulating the sympathetic nerves [37] including via exercise [38] or isoproterenol infusion [39, 40]. Many reports have found that the intestinal autonomic nervous system is closely related to the anterior cingulate cortex (ACC), thalamus, and/or PAG [41, 42]. In addition, the brain axis has been found to be important in regulating the pathology and physiology related to the heart. The parasympathetic preganglionic fibers of the heart originate from the nucleus ambiguous and dorsal motor nucleus of the brainstem. The sympathetic preganglionic fibers of the heart mainly originate from the regulation of higher centers such as the subthalamic nucleus and the periaqueductal gray matter. Our findings also provide evidence for the above point of view (Fig 4). We further hypothesized that the LPAG-Me5-Acs5-IML nerve loop may be a potential target for the integration of the sympathetic nerves of the small intestine and the heart in the central nervous system (Fig 6). This is based on the following lines of evidence: PAG has autonomic nerve regulation function, VlPAG has parasympathetic function [43], and LPAG has sympathetic function [44, 45]. The sympathetic fibers of LPAG project to Me5 and terminate at 5Adi [46]. A recent study shows that 5ADi has general visceral efferent fibers that send parasympathetic signals to the heart [47]. In addition, they can innervate gastrointestinal smooth muscles [48]. Here, we found that the heart and small intestine simultaneously receive nerve projections from LPAG, Me5, and 5Adi, which is supported by the observation of cardiac preganglionic nerve fibers and small intestinal preganglionic nerve fibers in the IML of the spinal cord.

## Limitations

This study has several technical limitations. Firstly, the neural loop was analyzed using the tendency of PRV to invade neurons. However, some debate about the specificity of virus transmission between neurons is still unavoidable. In this regard, it is worth noting that virus cleavage and non-synaptic release of viruses into the extracellular environment may destroy neural loops, thus hindering the transport of viruses by neural loops. Secondly, because of its toxicity, the PRV injected into different peripheral sites in this experiment is enough to kill mice within 110 h. As a result, some other related areas may not be labeled by the fluorescent protein. Thirdly, in future studies, experiments should be further conducted to determine the types of co-labeled neurons.

## Supporting information

S1 Video (DOCX)
