# Supplementary material for: Study on the central neural pathway and the relationship between the heart and small intestine via a dual neural tracer
Source: PLoS One. 2022 Nov 22;17(11):e0277644. doi: 10.1371/journal.pone.0277644 (PMC9681100; doi:10.1371/journal.pone.0277644)
Supplement: S1 Video — (DOCX) [file pone.0277644.s001.docx]

Supplementary Video 1 <https://figshare.com/s/df5171d4f2bd8ed97ddd> DOI：[10.6084/m9.figshare.15098250](https://doi.org/10.6084/m9.figshare.15098250)
